# Rapid Induction of Neural Differentiation in Human Umbilical Cord Matrix Mesenchymal Stem Cells by cAMP-elevating Agents

**Published:** 2016-09-13

**Authors:** Atefeh Shahbazi, Majid Safa, Fatemeh Alikarami, Saeid Kargozar, Mohammad Hossein Asadi, Mohammad Taghi Joghataei, Mansoureh Soleimani

**Affiliations:** 1 *Department of Stem Cells and Developmental Biology at Cell Science Research Center, Royan Institute for Stem Cell Biology and Technology, ACECR, Tehran, Iran.*; 2 *Cellular and Molecular Research Center, Iran University of Medical Sciences, Tehran, Iran.*; 3 *Department of Hematology, Faculty of Allied Medicine, Iran University of Medical Sciences, Tehran, Iran.*; 4 *Department of Tissue Engineering and Applied Cell Sciences, School of Advanced Technologies in Medicine, Tehran University of Medical Sciences, Tehran, Iran.*; 5 *Department of Anatomy, School of Medicine, Baqiyatallah University of Medical Sciences, Tehran, Iran.*

**Keywords:** Umbilical cord matrix mesenchymal stem cells, neural differentiation, cAM

## Abstract

Human umbilical cord matrix (hUCM) is considered as a promising source of mesenchymal stem cells (MSCs) due to several advantages over other tissues. The potential of neural differentiation of hUCM-MSCs is of great interest in the context of treating neurodegenerative diseases. In recent years, considerable efforts have been made to establish *in vitro* conditions for improving the differentiation of hUCM-MSCs toward neuronal cells. In the present study, we evaluated the neural differentiation potential of hUCM-MSCs in the presence of cAMP-elevating agents forskolin and 3-isobutyl-1-methylxanthine (IBMX). hUCM-MSCs were isolated from fetal umbilical cord and characterized by ﬂow cytometry analysis for mesenchymal specific markers. Mesodermal differentiation potential was assessed through selective media with lineage-specific induction factors. For assessment of neural differentiation, cells were cultured in the presence of cAMP-elevating agents for 8 and 24 h. The neuronal differentiated MSCs were characterized for neuronal specific markers by immunocytochemistry and western blotting. Isolated hUCM-MSCs were found positive for mesenchymal markers (CD73, CD90, and CD105) while negative for hematopoietic markers (CD34 and CD45) .Following neural induction, most cells represented neural-like cells morphology. Neural markers including β-tubulin III (Tuj-1), neuron-specific enolase (NSE), microtubule-associated protein-2 (MAP-2) and nestin were expressed in treated cells with respect to control group. The astrocyte specific marker, glial fibrillary acidic protein (GFAP) was also shown by immunofluorescence in treated cells.** (**These findings demonstrate that hUCM-MSCs have the ability to rapidly differentiate into neural cell types of neuron-like cells and astrocytes by cAMP-elevating agents without the presence of growth factors.

Mesenchymal stem cells (MSCs) are multipotent non-hematopoietic stem cells which have the capacity to differentiate into several mesenchymal lineages such as osteocytes (1), chondrocytes (2) and adipocytes (3). Due to their easy accessibility, straightfor-ward procedures of isolation and immunoregulatory property, MSCs are good candidates to be used in cell therapy (4). It has been reported that MSCs can also transdifferentiate into other cell types derived from germ layers including cardiomyocytes (5), hepatocytes (6) and neuro-cytes (7). In particular, several studies have shown the differentiation potential of MSCs into neural-like cells (8, 9). The use of MSCs for treatment of neurodegenerative diseases has become of interest. The potential application of MSCs in neurodegenerative diseases is based on their transdifferentiation capability into neural cells (10) in addition to their neuroprotective and immunoregulatory properties (11). Therefore, it is not surprising that the clinical applications of these stem cells for Parkinson’s disease (12), multiple sclerosis (13), Alzheimer's disease (14), amyo-trophic lateral sclerosis (ALS) (15) and Huntington's disease will increase in the coming years (16). 

The mesenchymal stem cells can be classified into two categories: MSCs derived from adult tissues such as bone marrow (17), adipose tissue (18), endometrial polyps (19) and MSCs derived from fetal/perinatal tissues such as placenta (20), amniotic membrane (21), umbilical cord blood and matrix (22). The common source of human MSCs is bone marrow (BM-MSCs); however, BM-MSCs comprise very small fraction (0.001% to 0.01%) of the total population of nucleated cells in the marrow and their proliferative capacity and differentiation potential decrease with age (23). Therefore, finding alternative sources of MSCs would be beneficial for both therapeutic and research purposes. Umbilical cord matrix (also known as the Wharton’s jelly) is emerging as a promising source of MSCs obtained by non-invasive methods. In contrast to BM-MSCs, umbilical cord matrix-MSCs (UCM-MSCs) have faster proliferation and greater *ex vivo* expansion capacity that might be due to the expression of telomerase by these cells (24). In addition, UCM-MSCs are more primitive than mesenchymal stem cells derived from other tissues and have the ability to remain undifferen-tiated for at least 10 passages *in vitro* (25). Interestingly, transplantation of UCM-MSCs is not associated with teratoma formation despite the primitive features of these cells (26). Thus, the umbilical cord matrix represents a pro-mising source of MSCs for stem cell-based therapies.

The second messenger molecule cyclic adenosine monophosphate (cAMP) is an important intracellular signaling mediator. Its formation is promoted by adenylyl cyclase activation that occurs after G-protein-coupled receptors are ligated by ligands, such as hormones, prostaglandins, and pharmacologic agents (27). The cAMP signaling pathway plays a critical role in many cellular functions including metabolism, cell differentiation, and apoptosis (28). Forskolin is an activator of adenylyl cyclase and IBMX inhibits the phosphodiesterase mediated degradation of cAMP to AMP, resulting in increased cAMP levels within the cell (39). In the present study, we isolated MSC from umbilical cord matrix and assayed the capacity of UCM-MSCs to differentiate *in vitro* into neural-like cells upon exposure to combination of cAMP-elevating agents IBMX and forskolin. We found that IBMX and forskolin induce neural-like cell morphology and enhance general neural markers like nestin, β-tubulin III (Tuj-1), neuron speciﬁc enolase (NSE), and microtubule-associated protein 2 (MAP2). Our data suggest that the elevation of intracellular cAMP plays a key role in the neural differentiation of UCM-MSCs.

## Materials and methods

**Isolation and culture of UCM-MSCs**

After the approval of the study protocol by the Medical Ethics Committee of the Iran University of Medical Sciences (IUMS), umbilical cord samples were obtained from Shariati Hospital following normal deliveries, with written informed consent of the parent(s). We used 3 independent umbilical cord units. Each umbilical cord unit was rinsed several times with sterile PBS (Sigma, St Louis, MO, USA) and cut into 4 cm lengths. To isolate UCM-MSCs, the cord blood was drained and clots flushed from the vessels. Next, the vessels were stripped completely from cord segments, the wall of the cord was opened and the tissue was chopped into 3–4 mm pieces. The fragments were then immersed in culture medium containing 0.1% collagenase type I (Sigma, USA) for 3 h and then 2% dispase (Gibco, Grand Island, NY, USA) for 30 min with gentle agitation at 37 °C. The digested tissues were filtered and washed 2 or 3 times using sterile PBS. The pellet was resuspended in Dulbecco’s Modified Eagle’s Medium with low glucose (DMEM-LG; Gibco, USA) supplemented with 10% fetal bovine serum (FBS; Gibco, USA), 100 U penicillin/streptomycin (Sigma, USA), and 2 mM L-glutamine (Gibco, USA) and seeded in non-coated T-25 cell culture flasks (Beckon Dickinson, San José, CA, USA). The cultures were maintained in a humidified atmosphere with 5% CO2 at 37 °C. After 3 days of culture, the non-adherent cells were removed by changing the medium. The cells were passaged and expanded when they had grown to 80% to 90% confluence. All experiments were carried out by MSCs between passages 1 to 3.

**Immunophenotype analysis of UCM-MSCs**

Flow cytometry (FACSort, BD, USA) was used to assess the immunophenotype of UCM-MSCs. Phycoerythrin (PE) conjugated antibodies against CD105, CD90, CD73 and   fluorescein isothiocyanate (FITC) conjugated antibodies against CD34 and CD45 were purchased from Becton Dickinson (CA, USA). 2×10^5 ^trypsinized cells were resuspended in 2% bovine serum albumin and were then incubated for 1 h in darkness with specific antibodies. After staining, the expression of the CD markers was analyzed by flow cytometry using Cell Quest Software (BD Bioscience, USA). Appropriate isotype controls were used in the experiments. 

***In vitro*** **differentiation of UCM-MSCs**

**Osteogenesis**

UCM-MSCs (third passage) at 60% conflu-ence were cultured in osteogenic differentiation medium supplemented with 90% α-MEM (Gibco, USA), 10% heat-inactivated FBS (Gibco, USA), 50 μg/mL ascorbic acid (Sigma, St. Louis, MO, USA), 5 mM β-glycerophosphate (Sigma, USA), and 100 nM dexamethasone (Sigma, USA) for 3 weeks. This culture medium was replaced every 2-3 days. Negative control was obtained by omitting osteogenic induction medium. To assess osteo-genesis, cells were stained by Alizarin Red S (Sigma Chemical Co) staining as an indicator of calcium deposits.

**Adipogenesis**

At 70% confluence, UCM-MSCs (third passage) for 14 days were cultured in adipogenic differentiation medium supplemented with 0.5 µM IBMX (Sigma, USA), 50 µM indomethacin (Sigma, USA), 0.5 µM dexamethasone (Sigma, USA), and 5 µg/mL insulin (Sigma, USA) and the medium was changed every 2-3 days. As a negative control, the normal UCM-MSCs were cultured in non-inducing medium. To evaluate adipogenesis, the cells were stained by Oil-red O (Sigma Chemical Co) staining and cytoplasmic lipid vacuoles were observed by light microscopy (Olympus DP70, Japan). 

**Chondrogenesis**

UCM-MSCs cultured in DMEM from passage 3 were harvested and centrifuged at 500g for 10 min. The resulting pellet was cultured in polypro-pylene tube with chondrogenic differentiation medium for 21 days. The medium was composed of 10^−8^ M dexamethasone (Sigma, USA), 5 mg/mL ascorbic acid 2-phosphate (Sigma, USA), 10 mM β-glycerophosphate (Sigma, USA), and 10 ng/mL transforming growth factor-β3 (PeproTech, Rocky Hill, NJ). The medium was replaced every 2-3 days. For the negative control the normal UCM-MSCs were cultured in DMEM-LG containing 10% FBS. After 3 weeks, the pellets were embedded in paraffin and sliced into 3 µm sections. To detect chondrogenesis, the sections were stained with toluidine blue and observed by light microscopy (Olympus DP70, japan).

**Neural induction of UCM-MSCs**

For neural induction, UCM-MSCs (passage 3) were cultured in DMEM medium until the cells reached 70-80% confluence. Medium was then removed and UCM-MSCs were incubated in DMEM-LG containing 10% FBS with 10 µM forskolin (Sigma, USA) and 200 µM 3-isobutyl-1-methylxanthine (IBMX; Sigma, USA); for 8 to 24 h at 37 °C, 5% CO2 and 95% humidity. As a control group, UCM-MSCs were cultured in DMEM-LG medium containing only 10% fetal bovine serum. For consistency of results, all tests were done in triplicate. 

**Immunocytochemistry analysis**

After neural induction for 8 to 24 h, the cells were fixed with 4% paraformaldehyde (PFA). The fixed cells were blocked with 10% goat serum (Vector Laboratories, Inc, Burlingame, CA, USA) for 20 min at room temperature. Afterward, the cells were incubated with primary antibodies, anti-mouse nestin (Chemicon, Temecula, CA, USA, 1:100), anti-rabbit neuron-specific class III β-tubulin (Tuj-1, Millipore, Billerica, MA, USA 1:100), anti-mouse microtubule associated protein-2 (MAP2, Chemicon, 1:200), anti-mouse neuron specific enolase (NSE, Dako, Carpinteria, CA, USA, 1:100) and anti-rabbit glial fibrillary acidic protein (GFAP, Chemicon, 1:300) overnight at 4°C. After PBS washing, FITC conjugated secon-dary antibodies (Molecular Probes, Invitrogen Co, CA, USA) were added and incubated at room temperature for 1 h. For nuclear staining, cells were treated with 1 μg/ml 4′,6-diamidino-2-phenylindole (DAPI) for 5 min at room temperature. Finally, cells were observed under fluorescence microscope (Zeiss, Germany). 

**Protein extraction and western blot analysis**

For total protein extraction, cells were washed three times with ice-cold PBS and then scraped off with a cell scraper. After centrifugation, cell pellets were collected into a 1.5 mL tube and lysed using radioimmunoprecipitation assay (RIPA) buffer [1% triton X-100, 1% sodium deoxycholate, 50 mM NaCl2, 50 mM tris-HCl, 1 mM sodium vanadate, 2 mM phenylmethanesulfonyl fluoride (PMSF)]. The lysed cells were centrifuged at 15,000g for 30 min at 4 °C. The supernatant containing proteins was quantified with Bradford assay solution (Bio-Rad). The sample was boiled for five min and used directly or stored at -80 °C until further processing. After electrophore-sis separation, transfer buffer (25 mM Tris base, 0.2 M glycine, 20% methanol) was used to transfer proteins to nitrocellulose membrane (Amersham Bioscience, USA) for 1 h under 30 V. The membrane was then treated with blocking buffer (5% skim milk, 1X TBS, 0.1% TWEEN 20) for 1 h and incubated with primary antibodies, nestin mouse monoclonal ( Abcam, 1:5000 in 1x TBS-T ), neuron-specific class III β-tubulin mouse monoclonal (Tuj-1, Abcam, 1-2µg/ml in 1x TBS-T), neuron specific enolase rabbit monoclonal (NSE, Abcam, 1:5000 in 1x TBS-T), and rabbit monoclonal phospho-CREB overnight at 4 °C. TBS-T washing buffer [1X TBS, 0.1% TWEEN 20] was used for 5 min three times. Afterward, anti-rabbit or anti-mouse HRP-linked secondary antibody (Dako, 1:2000 in 1x TBS-T) was added, incubated for 1 h and washed three times with TBS-T. To visualize speciﬁc antibody binding, the membrane was placed in a film cassette, exposed to chemiluminescence (ECL, Amersham Bioscience, USA) reagent and X-ray ﬁlm (Thermo Scientiﬁc) for various time periods. 

## Results

**Isolation and characteristics of MSCs derived from human umbilical cord matrix**

**Fig 1:**
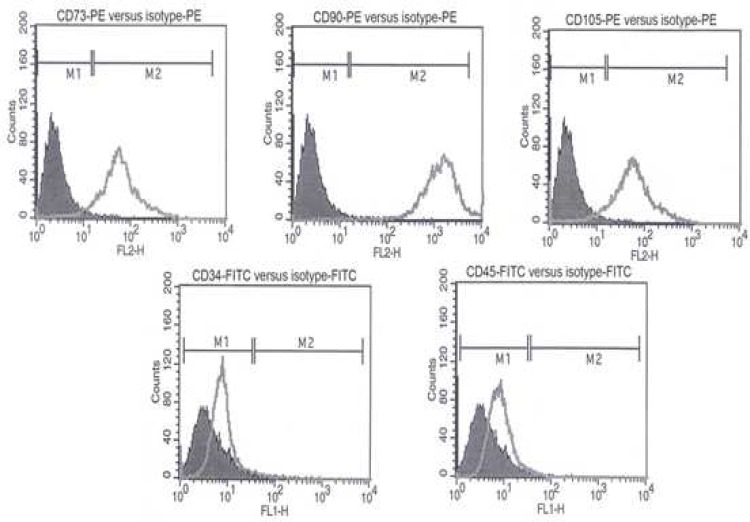
Immunophenotypic characterization of hUCM-MSCs. Cells at passage 3 were labeled with FITC- and PE-conjugated antibodies and analyzed by flow cytometry. Cells were strongly positive for CD73, CD90 and CD105 but negative for hematopoietic markers CD34 and CD45

MSCs were obtained from 3 distinct human umbilical cord matrix according to the enzymatic digestion method. After 1 week they became adherent cells, more uniform and were fibroblastic in shape. The expression of UCM-MSCs surface markers was analyzed by flow cytometry. Consistent with the previous reports (1, 29), these cells were uniformly positive for mesenchymal markers (CD73, CD105, and CD90) and were found negative for hematopoietic stem cell markers (CD34, CD45) (Figure 1).

***In vitro*** **differentiation potential of hUCM-MSCs**

**Fig 2:**
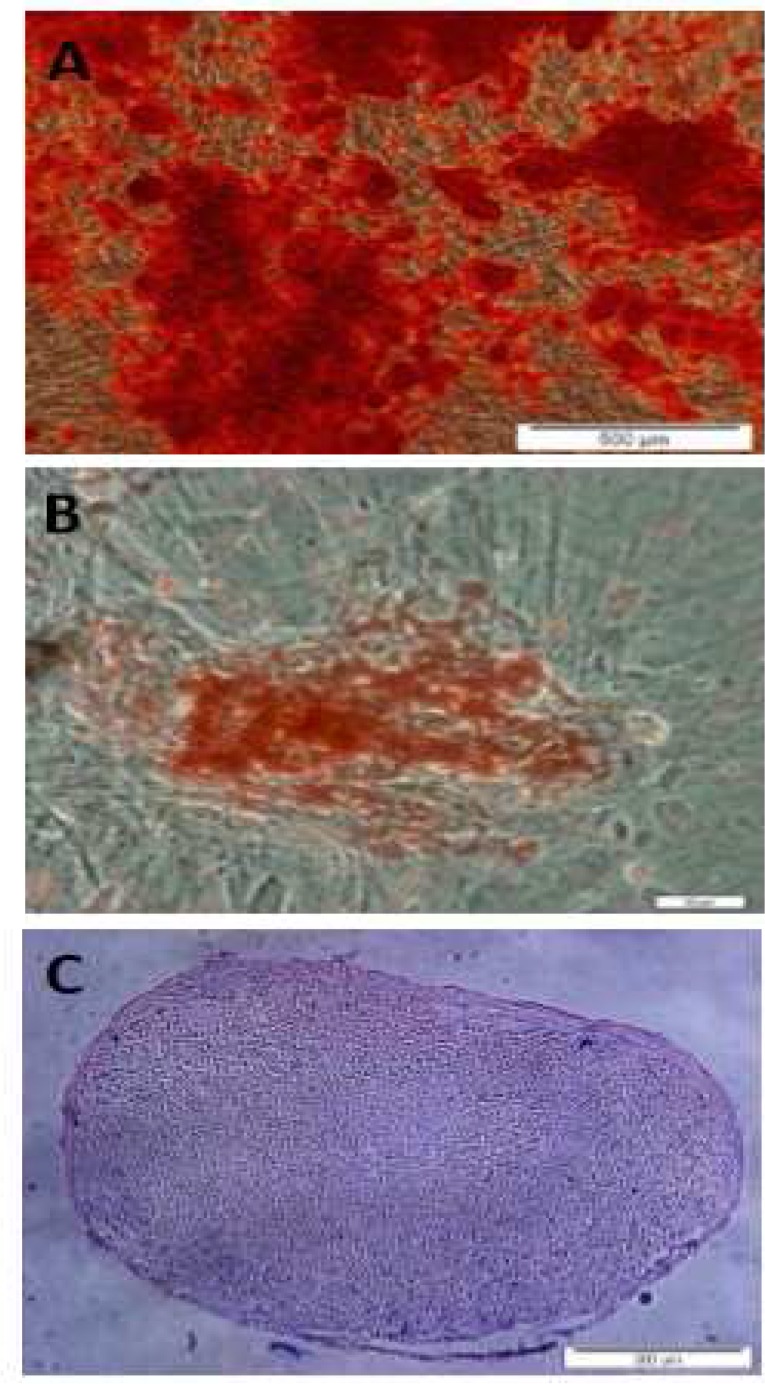
Mesodermal three- lineage differentiation of hUCM-MSCs. Cells at passage 3 were cultured for 21 days in selective media for osteogenic, adipogenic and chondrogenic differentiation. (A) Osteogenic differentiation was examined by Alizarin red S staining. (B) Adipogenic differentiation was shown by oil red O staining. (C) Chondrogenic differentiation was analyzed by toluidine blue O staining

To determine whether UCM-MSCs are able to differentiate into mesodermal cell types, the cells were cultured in selective media. The differentiation capacity of these cells into adipocytes, osteoblasts and chondrocytes were verified by specific staining (Figure 2).

**Fig 3:**
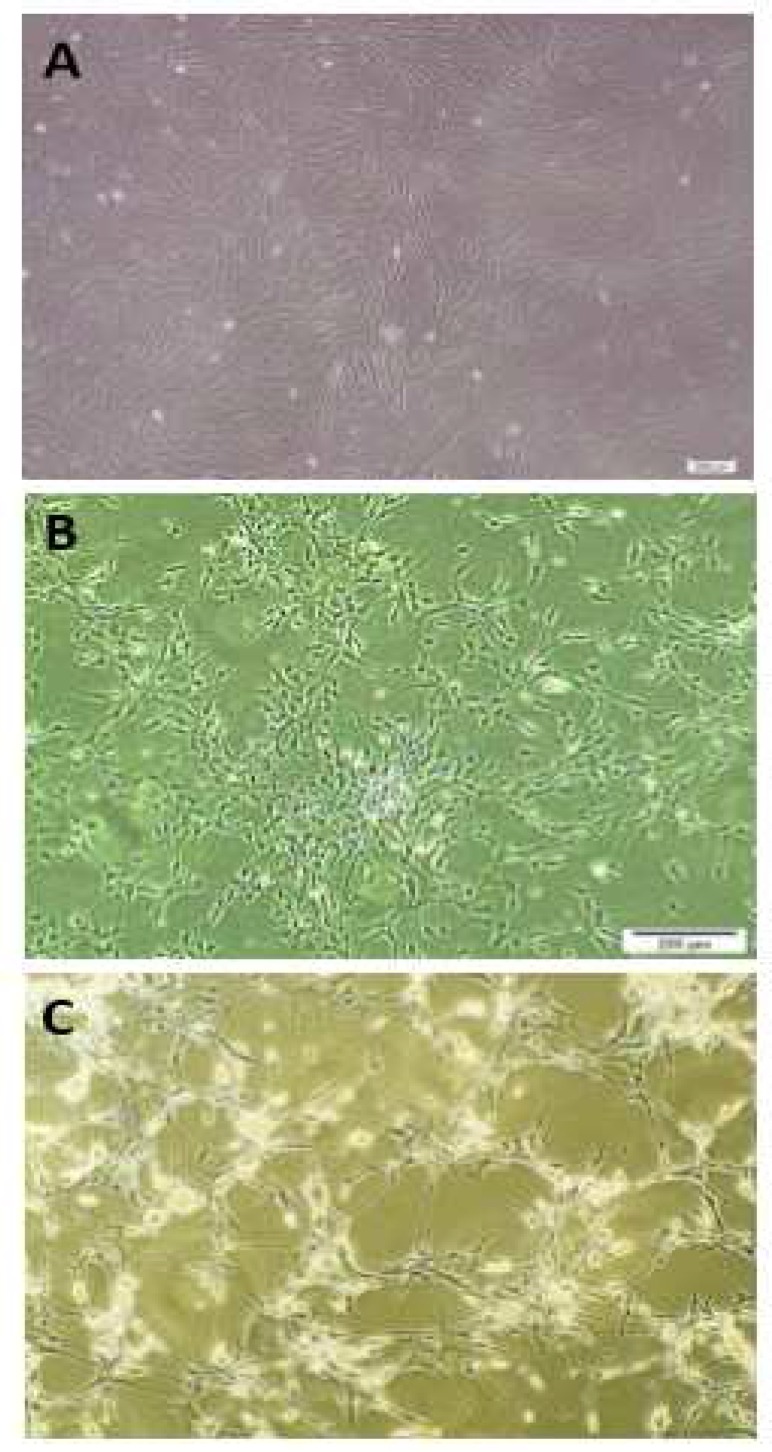
Neural differentiation and morphological changes. (A) The untreated hUCM-MSCs at passage 3 were plastic adherent with spindle shaped morphology. (B) Cell bodies were created after 8 h induction. (C) Typical neuron-like morphology was observed after 24 h treatment with forskolin and IBMX

After 21 days in osteogenic culture medium, osteogenesis was evidenced by the formation of mineralized nodules and calcium deposits, and confirmed by staining with Alizarin Red (Figure 2A). After 21 days of the adipogenic culture medium, the formation of intracellular lipid droplet in adipocytes was confirmed by Oil red O staining (Figure 2B).

Chondrogenic differentiation was assessed by toluidine blue staining of pellet sections after 21 days, whereby cartilaginous extracellular matrix will stain purple (metachromasia) while undiffer-entiated or fibrous tissue will stain blue (Figure 2C).

**Neural differentiation and morphological changes**

MSCs were isolated from human umbilical cord matrix incubated with neural induction factors (IBMX and forskolin) for 8 to 24 h. The early morphological changes were observed after 8h (Figure 3B) and typical neuron-like morphologies (including the retractile appearance of the cells, the presence of either bipolar or multipolar neurite-like projections and the presence of other secondary extensions) were observed after 24 h (Figure 3C) by phase/contrast microscopy. 

**Expression of neural markers using immu-nocytochemistry**

**Fig 4:**
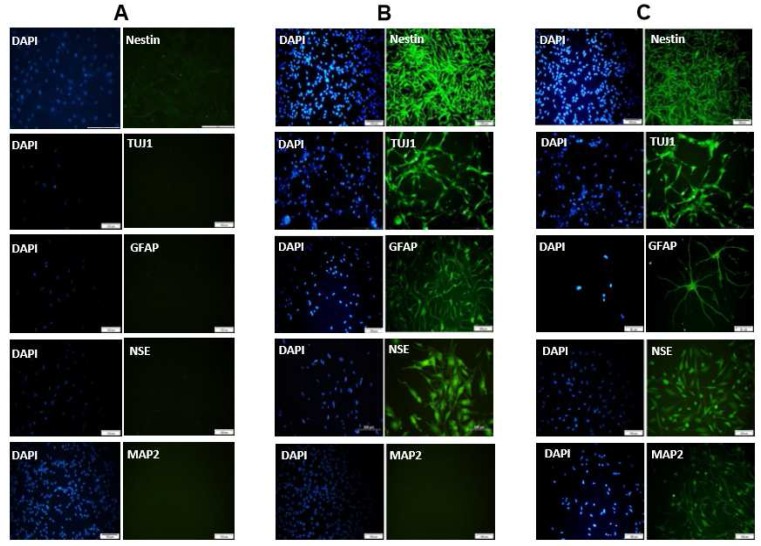
Immunocytochemistry analysis for neural differentiation markers in forskolin plus IBMX induced hUCM-MSCs. A: untreated hUCM-MSCs, all the neural markers (nestin, Tuj-1, GFAP, NSE and MAP2) were negative for un-treated hUCM-MSCs. B: hUCM-MSCs treated with forskolin and IBMX for 8 h were significant positive for the neural markers: nestin, b-tubulin III (Tuj-1), glial ﬁbrillary acidic protein (GFAP), neuron speciﬁc enolase (NSE), but negative for microtubule associated protein (MAP2). C: after 24 h incubation, expression of the neural markers (nestin, Tuj-1, GFAP, NSE and MAP2) was strongly expressed in forskolin plus IBMX induced hUCM-MSCs. The nucleus was stained with DAPI. Data are representative of three independent experiments

To verify neural differentiation, after 8 to 24 h treatment with combination of forskolin and IBMX, the cells were subjected to immunocytochemistry analysis. The result of immunocytochemistry revealed that neural markers (nestin, Tuj-1, NSE and MAP2) except MAP2 were positive after 8 h treatment. Interestingly, astrocyte specific marker (GFAP) was also expressed (Figure 4B). However, all the mentioned markers were expressed after 24 h (Figure 4C). None of the neural markers (nestin, Tuj-1, GFAP, NSE and MAP2) were expressed in undifferentiated hUCM-MSCs (Figure 4A).

**Expression of neural markers using western blot analysis**

**Fig 5:**
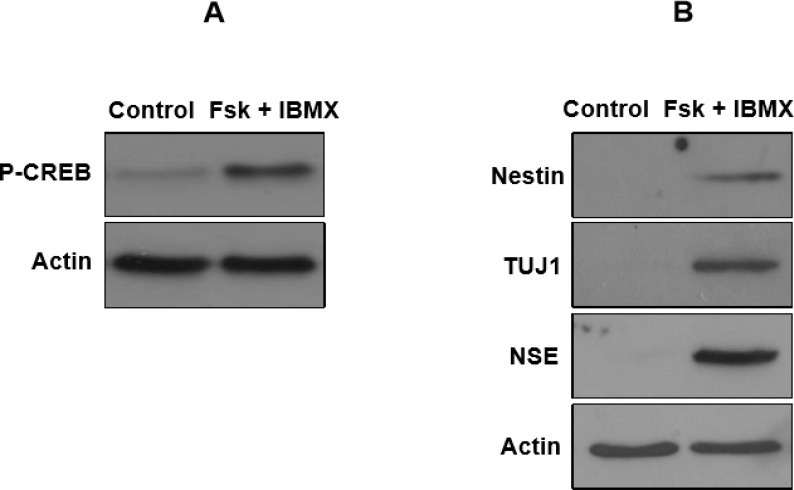
Western blot analysis for detection of specific neural markers in forskolin plus IBMX treated hUCM-MSCs. A: hUCM-MSCs were treated with cAMP-increasing agents for 24 h and western blot analysis was then performed for assessment of CREB phosphorylation. B: after 24 h of neural induction, western blot analysis demonstrated that the nestin, Tuj-1 and NSE were expressed in forskolin plus IBMX treated group respect to control. The intensity of each protein band was normalized against β-actin. Data are representative of three independent experiments

It is believed that cAMP-dependent protein kinase A (PKA) is the main effector for cAMP signal in eukaryotic cells (30). cAMP-mediated PKA activation induces cAMP-response element-binding protein (CREB) phosphorylation (31, 32). Thus, to indicate the role of cAMP levels in neural differentiation of hUCM-MSCs, a phospho-specific antibody was used to examine the phosphorylation of CREB at Ser-133. As presented in Figure 5A, combination of forskolin/IBMX enhanced the phosphorylation of CREB at Ser133 which is in support of the action of cAMP-elevated levels in neural differentiation of hUCM-MSCs. To confirm the cAMP-mediated induction of neural markers in hUCM-MSCs, western blot analysis was performed on hUCM-MSCs treated with or without combina-tion of cAMP-increasing agents forskolin and IBMX. After 24 h treatment, we found that the expression levels of nestin, Tuj-1 and NSE were higher in forskolin and IBMX-treated cells respect to control (Figure 5B). These findings indicate that cAMP-elevating agents can induce expression of neural proteins in hUCM-MSCs.

## Discussion

Stem cell-based therapies are promising approaches for the treatment of neurodegenerative diseases (12, 33). Recently, MSC has created major breakthroughs in the ﬁeld of regenerative medicine. hUCM is considered as a promising source of MSCs due to several advantages compared with other sources of MSCs (25). The potential of neural differentiation of hUCM-MSCs has generated great interest in the treatment of neurodegenerative diseases. Significant efforts have been made to investigate the appropriate approaches toward neural differentiation of hUCM-MSCs *in vitro* (34, 35). In the present study, we successfully isolated MSCs from human umbilical cord matrix. In agreement with previous studies (21, 26), these cells were adherent to plastic with spindle shaped morphology and revealed expression of mesenchymal markers (CD105, CD90, and CD73) but not hematopoietic markers (CD34 and CD45). In addition, they were able to differentiate *in vitro* into osteocytes, adipocytes and chondro-cytes. Sequentially, we used cAMP-increasing agents to induce neural differentiation in hUCM-MSCs. Several studies have reported that MSC derived from different human sources, could give rise to different types of neural cells *in vitro* by various agents such as a mixture of all-trans retinoic acid, forskolin, bFGF, PDGF, and heregulin-b1 (29) or a cocktail of edaravone, EGF and bFGF (35). In other studies it was shown that mixture of IBMX, db-cAMP, bFGF, all-trans retinoic acid and NGFβ (36) or neuroectodermal-induction medium supple-mented with N2 and EGF (37) also induced the neural differentiation of MSCs *in vitro*. In the current study, we have shown the neural differentiation of hUCM-MSCs by treatment with forskolin and IBMX in the absence of growth factors. Forskolin is an activator of adenylyl cyclase and IBMX inhibits the phosphodiesterase mediated degradation of cAMP to AMP, resulting in increa-sed cAMP levels within the cell (38). cAMP has been shown to play a trophic signal in the regenera-tion of the central nervous system. Many evidences indicate that cAMP can induce differentiation of MSCs into neural cells with expression of neural markers (39, 40). Jang et al. demonstrated that human adipose derived stem cells (hADSCs) differentiate into neuron-like cells *in vitro* using bFGF and forskolin (41). Our data showing that cAMP-elevating agents induce hUCM-MSCs diff-erentiation into neuron-like cells corroborate with the previous study of Zhang et al. (40). They have demonstrated early cAMP initiated neuron-like morphology changes and much later expression of neural markers in BM-MSCs (40). Our report provides evidence that activation of cAMP signaling pathway by combination of forskolin and IBMX in hUCM-MSCs results in neuron-like morphology and expression of neural markers as early as 8 h, and by 24 h, the cells also expressed mature neural marker MAP2. However, another study showed expression of neural markers by MSCs treated with various differentiation-inducing agents over a period of 24 h to several days (42). Interestingly, our experiment displayed negative expression of MAP2 within the first 8 h but positive expression after 24 h. This finding suggests that the longer incubation time with forskolin and IBMX has an important role in expression of mature neural marker. Expression of GFAP as a specific marker for astrocytes in forskolin/IBMX treated-hUCM-MSCs suggests that cAMP-elevating agents induce MSC differentiation into neural cell types of neuron-like cells and astrocytes.

Importantly, we found that none of the neural markers (nestin, Tuj-1, GFAP, NSE and MAP2) were expressed in naïve hUCM-MSCs. These results support the idea that the presence of neural markers in naïve hUCM-MSC may not be necessary for the ability of MSCs to differentiate into neural-like cells. Our findings are consistent with the previous study of Rong Zeng et al. that reported negative expression of MAP2, nestin, GFAP and NSE in undifferentiated BM-MSCs (35). A possible reason for the variations between our results and previous studies may be due to differences in the cell sources and neural induced agents that were applied. Our findings establish that hUCM-MSCs can differentiate into neural cells with expression of neural markers and morpho-logical changes in the presence of cAMP-elevating agents. To the best of our knowledge, this is the first study concerning human umbilical cord matrix derived MSCs induction into neuron-like cells in the presence of forskolin and IBMX without the use of growth factors. Further studies are required, however, to certify the safety and therapeutic dose of cAMP-elevating agents for neural differentiation of hUCM-MSCs.
